# Development of a Cognitive Assessment Battery for Adolescents and Adults with Kabuki Syndrome Type 1

**DOI:** 10.21203/rs.3.rs-9941281/v1

**Published:** 2026-08-03

**Authors:** Lauren Meier, Jacqueline Harris, Rowena Ng

**Affiliations:** Kennedy Krieger Institute; Kennedy Krieger Institute; Kennedy Krieger Institute

**Keywords:** KMT2D (lysine methyltransferase 2D), Kabuki syndrome (KS), visuospatial abilities, intellectual disability, executive functioning, clinical trial readiness, reliability

## Abstract

**Background:**

Kabuki syndrome type 1 (KS1) is a rare genetic neurodevelopmental disability syndrome characterized by significant weaknesses in visuospatial cognitive processes in comparison to other cognitive domains. There is a great unmet need for validated and specific cognitive outcome measures for clinical trials in patients with KS1. This study evaluated the validity and reliability of a Kabuki Syndrome Cognitive Assessment Battery (KS-CAB), a visuospatial assessment that is specific to KS1. Twenty-two individuals with molecularly confirmed KS1 (mean age = 21.1 years, range = 12.5–36.1 years) completed the same cognitive battery on two separate days.

**Results:**

Individuals with KS1 demonstrated consistent deficits in visuospatial cognition domains (often > 3 standard deviations below the mean), whereas other cognitive domains, such as language and executive functioning, were more variable. All visuospatial measures comprising the KS-CAB exhibited high test– retest reliability (Cronbach’s alpha = 0.81–0.97; intraclass correlation coefficient = 0.71–0.94). Subgroup analyses revealed that participants with missense variants outperformed those with truncating variants in visuospatial tasks.

**Conclusions:**

These findings are consistent with previous literature that visuospatial deficits are prominent in KS individuals and disproportionate to their intelligence quotient (IQ). In addition, this study demonstrated the reliability of 11 visuospatial subtests of existing cognitive measures, suggesting these are good candidates for clinical trial outcome measures in KS1.

## Background

Kabuki syndrome (KS) is a rare Mendelian disorder of the epigenetic machinery (MDEM) characterized by intellectual disability, distinctive facial features, fetal fingertip pads, postnatal growth deficiency, and skeletal abnormalities [1]. Approximately 60–80% of KS cases are caused by pathogenic variants in *KMT2D* (lysine methyltransferase 2D) [2], which is known as Kabuki syndrome type 1 (KS1) (MIM [Mendelian Inheritance in Man] #147920). *KMT2D* is a histone methyltransferase that adds a trimethylation mark to the fourth lysine of histone 3 (histone H3 lysine 4 trimethylation, or H3K4me3) [3]. When a pathogenic variant in *KMT2D* occurs, it disrupts the normal deposition of histone methylation marks, leading to altered chromatin regulation and downstream effects on gene expression [3]. Although KS1 presents with a broad range of clinical features, it is fundamentally a neurodevelopmental disability syndrome. While initial reports of KS described the cognitive profile broadly as mild to moderate intellectual disability, more recent studies have better characterized the neurocognitive phenotype [4–6]. Individuals with KS have specific weaknesses in visuospatial perception, reasoning, memory, and visuomotor integration, with relative strengths in language skills and variable executive functioning skills [5, 7]. These visuospatial weaknesses are apparent compared with an individual’s other cognitive skills as well as compared with IQ-matched controls without KS [4–6].

Several studies on mouse models of KS1 have shown correction of the cognitive phenotype with treatments [8–10]. Thus, there is promise for the treatability of intellectual disability in KS1. For use in clinical trials, cognitive outcome measures must demonstrate reliability and validity across repeated administrations, sensitivity to disease-relevant impairment, and sufficient variability. This study aims to establish the validity and reliability of certain subtests for inclusion in a visuospatial-focused cognitive battery, the Kabuki Syndrome Cognitive Assessment Battery (KS-CAB), for adolescents and adults with KS1 that can potentially be used as an outcome measure for future clinical trials.

## Methods

### Recruitment

Twenty-two individuals with KS1 participated in this study (15 Female, 7 Male, mean [M] age = 21.1; standard deviation [SD] = 7.09; range = 13.6–36.1; see Table 1). Participants were recruited from clinic populations at Johns Hopkins and Kennedy Krieger Institute and from patient groups (e.g., Kabuki Syndrome Foundation) with the use of advertisements on websites or social media. All individuals carried a pathogenic or likely pathogenic variant in *KMT2D* and met the diagnostic criteria for KS1 [11], confirmed by a physician with clinical expertise in KS. Additionally, we only included participants older than 13 years because the goal was to test a consistent battery in measures that had almost all norms in adolescents to adults. The majority of individuals were non-Hispanic white (72.73%) (Table 1). Among the 22 participants, nine had a frameshift variant, seven had a missense variant, four had a nonsense variant, and two had splice site variants (Table 1). This study was approved by the Institutional Review Board at Johns Hopkins University School of Medicine. All participants completed a written consent form prior to testing.

### Cognitive assessment battery

The KS-CAB was designed to prioritize measures that index visuospatial perception, visuospatial construction, and visuomotor integration, as well as select aspects of executive functioning, as these areas were previously identified as uniquely at risk in affected individuals [5, 7]. Subtests were drawn from established neuropsychological instruments and selected based on their ability to (1) operationalize visuospatial constructs known to be impaired in KS1, (2) span a wide range of performance levels within the affected population, and (3) be feasibly administered across adolescence and adulthood. Measures of general intellectual ability and language were included for descriptive and correlational purposes but were not intended to serve as primary outcome measures within the KS-CAB. Participants completed the KS-CAB twice on consecutive days (referred to as Day 1 and Day 2) at Kennedy Krieger Institute.

The Visual Perceptual (VP) and Visual-Motor Integration (VMI) tests of the Beery-Buktenica Developmental Test of Visual-Motor Integration 6th Edition (VMI-6) assess visuomotor and visual perception skills [12]. The Brief Visuospatial Memory Test (BVMT) [13] and Neuropsychological Assessment Battery Shape Learning (NAB-SHL) [14] assess visual memory. Both of these measures evaluate immediate and delayed recall, percent retention, and recognition. The Benton Judgment of Line Orientation (JLO) [15] measures visual perception through interpreting the orientation of lines in space. The Block Design (BD) subtests of the Wechsler Intelligence Scale for Children Fourth Edition (WISC-IV) [16] and the Wechsler Adult Intelligence Scale for Adults Fourth Edition (WAIS-IV) [17] were used to evaluate visual construction.

The National Institutes of Health (NIH) Toolbox Cognitive Battery (NIHTB-CB) is an iPad-based cognitive battery that assesses several areas of cognition [18] and has been validated in individuals with intellectual disability [19]. Dimensional Change Card Sort (DCCS) and Flanker Inhibitory Control and Attention (Flanker) both assess aspects of executive functioning, and Picture Sequence Memory (PSM) evaluates visual memory.

In addition to the KS-CAB, the WISC-IV and WAIS-IV were performed once to calculate full scale intelligence quotients (FSIQs) and assess language skills. It contains 10 subtests that evaluate cognitive functions and intellectual ability. The WISC-IV was administered to individuals 13–16 years old, whereas the WAIS-IV was administered to individuals older than 16 years.

### Data strategy

All statistical analyses were performed in R version 4.41. Standard scores were used for descriptive statistics to allow comparisons across ages, unless noted in the summary of group-level cognitive performance data in Table 2 (i.e., JLO, BVMT percent retention, BVMT recognition, and NAB). For test-retest reliability, raw scores were used. Cronbach’s alpha (α) and intraclass correlation coefficients (ICC) were computed (Table 3). Cronbach’s α greater than 0.75 was considered strong reliability between testing days. ICC less than 0.50 was considered poor reliability, 0.50–0.75 moderate, 0.75–0.90 good, and > 0.90 excellent. Standard scores were also used for correlations comparing visuospatial measures (Table 4) and to conduct t-tests for sex differences (Supplemental Table 1), unless a standard score was not available.

## Results

### Cognitive testing summary results

Group-level cognitive performance data are summarized in Table 2. Overall intellectual functioning fell within the mild intellectual disability range (FSIQ M = 69.5, SD = 13.1). Consistent with prior reports, participants demonstrated marked impairments in visuospatial perception, construction, learning, and visuomotor integration, with performance frequently more than three standard deviations below age-based norms. Severe deficits were observed on the BD, VMI, VP, JLO, and BVMT immediate recall. On the JLO, eight participants (36%) had a raw score of zero. On the BVMT, while nearly all participants scored at least a few raw points, 12 participants (60%) had the lowest possible scaled score of 20 on the immediate recall portion. In contrast, performance on measures assessing language (WAIS-IV/WISC-IV vocabulary, Similarities), attention, visual memory, and executive functioning (NIHTB-CB measures) were more variable and generally less impaired, typically falling 1–2.5 standard deviations below normative expectations.

**Figure 1:**
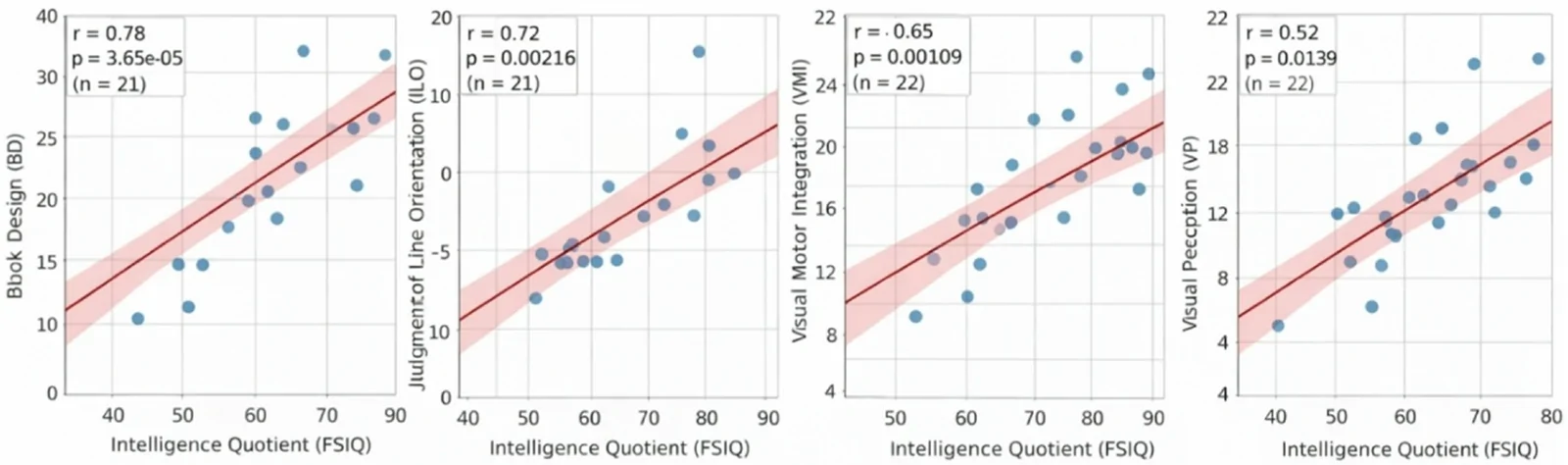
Associations between FSIQ score and visuospatial test performance in individuals with KS1.

**Figure 2:**
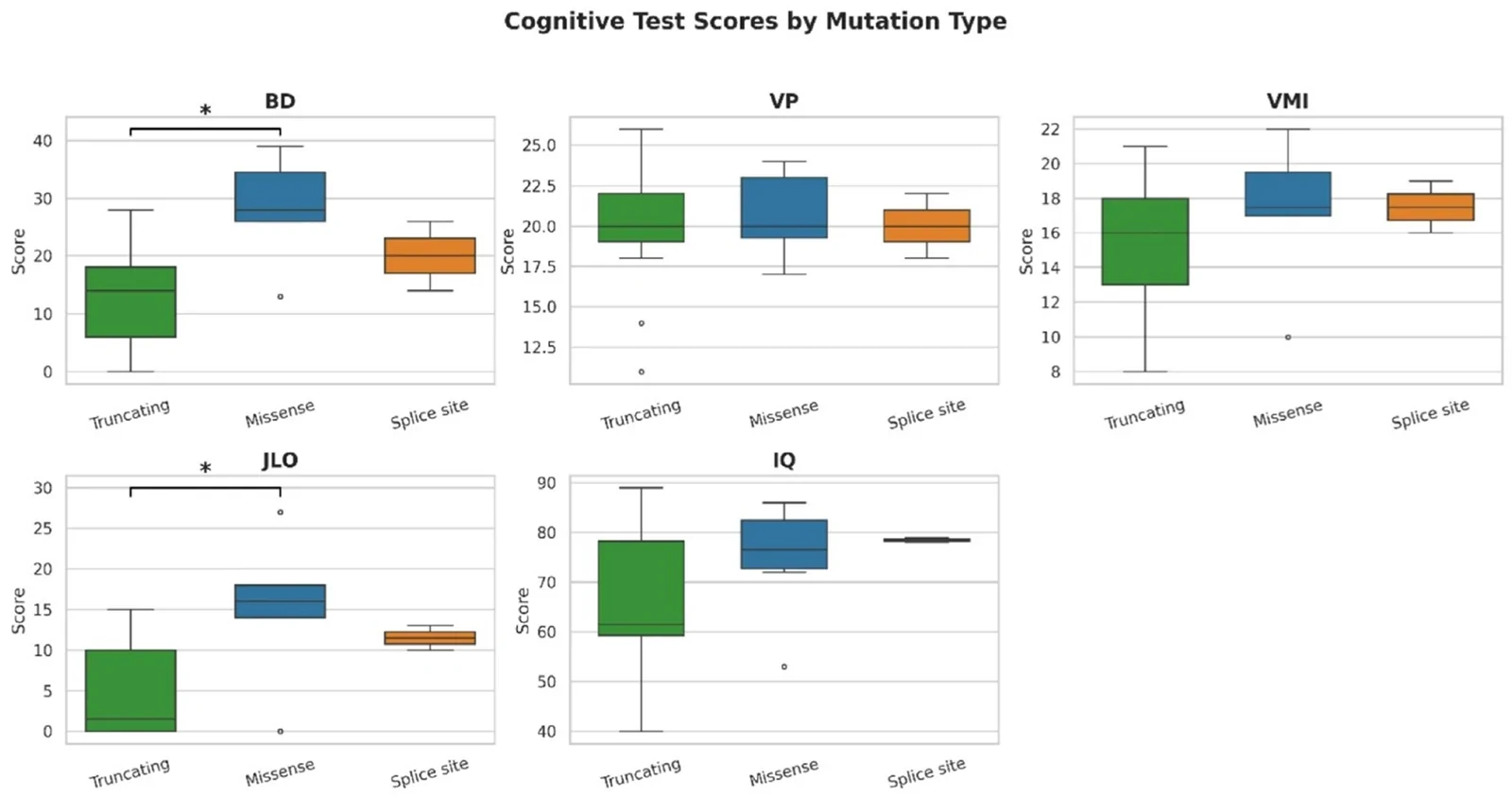
Comparison of visuospatial test and FSIQ performance by KMT2D mutation type (missense, truncating, or splice site).

Visuospatial measures were strongly associated with overall intellectual functioning (Fig. 1). The BD, JLO, and VMI all demonstrated strong correlations with the FSIQ (all r > 0.60). There was no significant effect of age on standardized scores across measures. Females performed significantly better than males on BVMT Delayed Recall only, no other sex effects were observed. Genotype–phenotype analyses revealed that individuals with missense variants outperformed those with truncating variants on BD (p = 0.015) and JLO (p = 0.027), whereas no genotype effects were observed for language or NIHTB-CB executive measures (Fig. 2).

### Test-retest reliability of the KS-CAB

The test–retest reliability across consecutive testing days is summarized in Table 3. All primary visuospatial measures demonstrated moderate to excellent reliability, with Cronbach’s α values ranging from 0.81–0.97 and corresponding ICCs ranging from 0.70–0.94 (see Table 3). BD, VMI, VP, and JLO, all of which are visuospatial measures, showed particularly strong stability across days (α ≥ 0.81; ICCs ≥ 0.80). Measures of visuospatial learning and memory (BVMT and NAB-SHL) showed evidence of modest practice effects, with higher mean scores on Day 2; however, reliability indices remained acceptable to strong for BVMT Delayed Recall (α = 0.93, ICC = 0.85), BVMT Immediate Recall (α = 0.94, ICC = 0.84), and NAB-SHL Forced-Choice Recognition (α = 0.87, ICC = 0.71). The PSM did show modest practice effects as well (α = 0.86, ICC = 0.73). Executive functioning measures from the DCCS and Flanker also demonstrated good test–retest reliability (α = 0.92, 0.86, ICC = 0.86, 0.71; respectively).

### Intercorrelations among visuospatial measures

Pairwise correlations using standard scores (unless only a raw score was available, see Table 2 note) among the Day 1 visuospatial measures are presented in Table 4. BD showed strong associations with the VMI (r = 0.67, p < 0.001) and JLO (r = 0.90, p < 0.001), and a moderate association with VP (r = 0.43, p = 0.051). Similarly, performance on the VMI was strongly correlated with that of JLO (r = 0.78, p < 0.001) and BVMT Immediate Recall (r = 0.76, p < 0.001) and moderately correlated with score on BD (r = 0.67, p < 0.001) and VP (r = 0.40, p = 0.065). VP showed weaker relationships with the other visuospatial measures (r = 0.30–0.50, p = 0.024–0.614). Together, JLO was strongly correlated with BD, VMI, and BVMT Immediate Recall (r = 0.70–0.90, all p < 0.001), whereas BVMT Immediate and Delayed Recall were highly correlated with each other (r = 0.89, p < 0.001). NAB-SHL Immediate Total showed moderate correlations with BD, VMI, and JLO (r = 0.54–0.64; p = 0.002–0.014) and a weak association with VP (r = 0.12, p = 0.614).

## Discussion

This study confirms previously published data that individuals with KS1 have specific weaknesses in visuospatial perception, reasoning, construction, learning, and visuomotor integration. These deficits align with preclinical findings that suggest dentate gyrus dysfunction in KS1 mouse models [8]. Visuospatial measures were strongly correlated with overall intellectual functioning, as indexed by FSIQ (Fig. 1). Such associations are expected in neurodevelopmental disability populations, as performance on complex problem-solving tasks necessarily contributes to global cognitive indices. However, the strength and consistency of correlations between FSIQ and multiple visuospatial measures in KS1, coupled with higher and more variable performance in language and executive functioning domains, suggest that visuospatial deficits may disproportionately contribute to global intellectual impairment in this syndrome. This finding supports the idea that a visuospatial composite battery captures the specific area of deficit for KS1, beyond just global intelligence. This study also adds to the existing body of literature by showing a genotype‒phenotype correlation in which participants with missense variants perform better on select measures of visuospatial perception than those with truncating variants, potentially indicating a dosage effect of KMT2D activity on visuospatial cognition.

Additionally, this study is the first to investigate the psychometric properties and utility of specific visuospatial cognition subtests as potential outcome measures in a future clinical trial in KS1. Outcome measures for clinical trials must (1) assess domains that are meaningfully impaired in the target population, (2) demonstrate adequate reliability across repeated administrations, (3) show sufficient variability to capture individual differences, and (4) minimize floor and ceiling effects that limit the interpretability of change over time. For this purpose, we investigated four measures of visuospatial perception and reasoning and visual motor integration (BD, JLO, VMI, and VP), three measures of visual spatial learning and memory (BVMT, NAB-SHL, and PSM), and two measures of executive function (Flanker and DCCS) to index cognitive domains of weakness specific to KS1 [5, 7].

Measures of visuospatial learning and memory (BVMT and NAB-SHL) demonstrated evidence of modest practice effects, with higher scores on Day 2 administrations. More importantly, performance on immediate recall trials was markedly impaired for most participants, likely reflecting severe difficulties in lower-order visuospatial perception and visuomotor integration. When initial learning is substantially compromised, interpretation of delayed recall or retention indices becomes ambiguous, as poor delayed performance may reflect deficits in perceptual encoding, learning, memory, or a combination of these processes. Accordingly, BVMT and NAB-SHL may be suboptimal outcome measures for isolating visuospatial memory change in KS1. The scores on the PSM were not significantly impaired in this population. PSM uses narrative to order the pictures, and it is unclear whether individuals with KS1 are performing well on this measure due to visuospatial memory or narrative memory. Either way, better performance also makes PSM a less desirable potential outcome measure if a therapy targets areas of deficit.

For the measures of visuospatial perception and reasoning and visual motor integration, on the JLO, more than one third of the participants received a raw score of zero, indicating significant floor effects and raising concerns that this test may not adequately capture the population variance in this cognitive function in KS. Although JLO demonstrated acceptable test–retest reliability, repeated floor-level scores provide limited information about individual variability or potential cognitive change over time. Given the strong correlations between JLO raw scores and performance on other visuospatial measures (e.g., BD, VMI), the visuospatial perception constructs indexed by JLO may be more effectively captured by alternative tasks that demonstrate greater variability and reduced floor effects in this population. In contrast, VMI, VP, and BD demonstrated a favorable combination of marked impairment, strong test– retest reliability, and a wide range of performance scores. These measures appear well-suited to capture both group-level deficits and individual variability within KS1, making them strong candidates for clinical trial outcome measures.

In addition to the visuospatial measures, which target the most consistently impaired domains in KS1, the two NIHTB-CB executive functioning measures (Flanker and DCCS) also showed group-level impairment but variability within the group and strong test-retest reliability. These measures are easy to administer and capture a different but overlapping cognitive domain and are suitable for inclusion into the KS-CAB.

Based on the data in this study, we propose that VMI, VP, BD, DCCS, and Flanker would all be excellent candidates for inclusion as individual outcome measures or as composite measures in future clinical trials in KS in adolescents and adults. This approach targets the most consistently impaired cognitive domains in KS1 while optimizing reliability, interpretability, and sensitivity for use in future intervention studies.

The current study has several limitations. First, we administered the battery to participants on two consecutive days. Despite strong test-retest reliability observed across measures, this study procedure may have contributed to potential practice effects, as evidenced in select measures. Second, we had a very small sample size with mostly truncating variants and white non-Hispanic sample, limiting the generalizability of findings. Future studies should include a larger and more diverse sample size, as well as more non-truncating variants. Third, this study had a restricted age range of participants, as the KS-CAB was evaluated only in individuals aged 13 years and older. While this age range is appropriate for many anticipated clinical trials in KS1, cognitive demands and measurement sensitivity may differ substantially in younger children. Widening the age range will be essential for developing a comprehensive and developmentally sensitive cognitive outcome framework for the entirety of the KS1 population.

## Conclusions

Visuospatial deficits are a core cognitive feature of KS1 and contribute significantly to overall cognitive functioning. This is the first study to systematically evaluate the reliability and clinical utility of specific visuospatial subtests as outcome measures for clinical trials in this population. The proposed KS-CAB includes the BD, VP, VMI, DCCS, and Flanker, all of which demonstrated strong test-retest reliability and validity. These subtests target the most impaired cognitive domains in KS1 individuals and show significant potential as outcome measures for future clinical trials. Future studies should evaluate its sensitivity to intervention-related change and expand validation across broader and more diverse KS1 populations.

## Supplementary Files

This is a list of supplementary files associated with this preprint. Click to download.

- SupplementaryTable11.docx

## Figures and Tables

**Table 1:** Participant characteristics and KMT2D variant information for the KS1 cohort.

ID	Sex	Age (years)	Inheritance	Mutation type	Variant	Identity
KS001	M	15.8	De novo	Frameshift	c.3190dupG	Black
KS002	M	28.8	De novo	Nonsense	c.14006C > G	White
KS003	F	36.1	De novo	Frameshift	c.14580dupT	White
KS004	M	22.7	De novo	Frameshift	c.5124_5125delAC	White
KS005	F	28	De novo	Missense	c.15536G > A	White
KS006	F	18.5	De novo	Frameshift	c.13432dupC	White
KS007	F	15.3	Unknown	Splice site	IVS50 + 5G > A	Asian
KS008	F	13.8	De novo	Frameshift	c.5278_5279delAA	Asian
KS009	M	20.3	De novo	Frameshift	c.13432dupC	White
KS010	F	30.3	De novo	Missense	c.15536G > A	White
KS011	F	21.4	De novo	Frameshift	c.7481_7482insT	White
KS012	F	22.8	De novo	Nonsense	c.10813 C > T	White
KS013	F	16.6	De novo	Frameshift	c.7643dupA	White
KS014	F	13.6	De novo	Missense	c.15251T > C	White
KS016	F	12.5	De novo	Nonsense	c.16018C > T	Hispanic
KS017	M	17.4	De novo	Missense	c.15142C > T	White
KS019	F	20.5	De novo	Missense	c.15626G > T	White
KS021	M	21.8	De novo	Missense	c.15143G > A	White
KS022	F	35.6	De novo	Nonsense	c.14641C >T	White
KS023	F	25.1	De novo	Frameshift	c.13681del	Asian
KS024	M	12.7	De novo	Missense	c.15535C > T	White
KS029	F	14	De novo	Splice site	c.8366 + 2T >A	White

**Table 2:** Cognitive testing in the KS1 group.

Tests applied	Domain covered	Number Completing Measure	Raw Score Mean (SD)	Range of Possible Raw Scores	Standard Score^1^ Mean (SD)	Range of Possible Standard Scores
Beery Developmental Test of Visual Motor Integration (VMI)	Visual motor integration	22	16.17 (3.64)	0–30	51.13 (8.9)	45–155
Beery Developmental Test of Visual Perception (VP)	Visuospatial perception	22	19.87 (3.48)	0–30	59.39 (15.23)	45–155
Benton Judgment of Line Orientation (JLO)^3^	Visuospatial perception	22	7.91 (7.42)	0–30	n/a	n/a
Brief Visuospatial Memory Test (BVMT) Immediate Recall^2^	Visuospatial learning, construction, memory	20	8.5 (7.07)	0–36	23.05 (5.58)	20–80
Brief Visuospatial Memory Test (BVMT) Delayed Recall^2^	Visuospatial construction and memory	20	3.35 (3.13)	0–12	23.21 (7.28)	20–80
Brief Visuospatial Memory Test (BVMT) Percent Retention	Visuospatial memory	20	70.7 (46.19)	0–100	n/a	n/a
Brief Visuospatial Memory Test (BVMT) Recognition Discrimination Index	Visuospatial memory	20	3.95 (2.42)	−6 to 6	n/a	n/a
Neuropsychological Assessment Battery Shape Learning (NAB-SHL) Immediate Total^4^	Visuospatial learning and memory	20	11.8 (5.28)	0–30	n/a	n/a
Neuropsychological Assessment Battery Shape Learning (NAB-SHL) Delay Total^4^	Visuospatial memory	21	4.14 (2.00)	0–10	n/a	n/a
Neuropsychological Assessment Battery Shape Learning (NAB-SHL) Forced-Choice Recognition	Visuospatial memory	21	4.27 (3.06)	0–10	n/a	n/a
NIH Toolbox Dimensional Change Card Sort (DCCS)^1^	Executive functioning – switching	11	27.64 (2.2)	0–30	70.82 (10.80)	40–160
NIH Toolbox Flanker Inhibition (Flanker)^1^	Executive functioning – inhibition	18	18.84 (2.24)	0–20	61.72 (6.87)	40–160
NIH Toolbox Picture Sequence Memory (PSM)^1^	Visual memory	18	11.16 (6.31)	0–30	91.78 (13.06)	40–160
Wechsler Adult Intelligence Scales - V or Weschler Intelligence Scales for Children - IV (WAIS-IV/WISC-IV) Full Scale Intelligence Quotient (FSIQ)^5^	General cognition	22	n/a	n/a	69.48 (13.14)	40–140
WAIS-IV/WISC-IV Vocabulary Subtest^5,6^	Language	22	23.87 (10.35)	0–44	6.27 (2.96)	1–19
WAIS-IV/WISC-IV Similarities Subtest^5,6^	Language	22	23.26 (7.28)	0–33/36^8^	6.78 (2.52)	1–19
WAIS-IV/WISC-IV Block Design (BD) Subtest^5,6^	Visuospatial construction	22	18.22 (10.36)	0–66/68^8^	4.48 (2.52)	1–19
WAIS-IV/WISC-IV Matrix Reasoning Subtest^5,6^	Abstract reasoning	22	13.91 (4.07)	0–26/35^8^	6.22 (3.29)	1–19
WAIS-IV/WISC-IV Digit Span Subtest^5,6^	Attention and memory	22	22.57 (7.38)	0–48/32^8^	7 (3.16)	1–19
WAIS-IV/WISC-IV Coding Subtest^5,6^	Processing speed	22	30.87 (14.95)	0–135/119^8^	3.61 (2.19)	1–19

*Note*:

1 Standard scores, with a mean of100 and standard deviation of 15, are outlined in the table column unless otherwise indicated.

2 T-scores used for standard scores

3 No standard scores are used because they differ for different age ranges

4 No standard scores because much of the study population is out of the age range

5 WAIS-IV or WISC-IV was used as appropriate for the age of participants

6 Scaled score used for standard scores

7 WAIS-IV total/WISC-IV total

**Table 3:** Mean raw scores (SD) for the Day 1 and Day 2 administrations and test–retest reliability (Cronbach’s α and ICC’s) for the KS-CAB subtests.

Tests	Day 1	Day 2		
	Mean (SD)	Mean (SD)	α	ICC
BD	18.22 (10.36)	19.19 (9.85)	0.97	0.94
BVMT Delayed Recall	3.35 (3.13)	3.85 (3.60)	0.93	0.85
BVMT Immediate Recall	8.5 (7.07)	10.75 (9.04)	0.94	0.84
BVMT Recognition Discrimination Index	3.95 (2.42)	3.8 (2.17)	0.71	0.38
JLO	7.91 (7.42)	9.43 (8.46)	0.85	0.88
NAB-SHL Immediate Total	11.8 (5.28)	12.6 (6.51)	0.8	0.66
NAB-SHL Delay Total	4.14 (2.00)	4.65 (1.79)	0.65	0.48
NAB-SHL Forced-Choice Recognition	4.27 (3.06)	4.8 (3.28)	0.87	0.76
DCCS	27.64 (2.20)	27.55 (1.38)	0.86	0.71
Flanker	18.84 (2.24)	18.83 (2.40)	0.92	0.86
NIHTB-PSM	11.16 (6.31)	10.71 (8.92)	0.86	0.73
VMI	16.17 (3.64)	16.36 (3.63)	0.9	0.83
VP	19.87 (3.48)	20.23 (4.33)	0.81	0.70

**Table 4:** Pearson correlation coefficients (r) and associated p values among Day 1 visuospatial measures in the KS1 cohort.

BD vs VMI	p	r
	0.001	0.670
BD vs VP	0.051	0.432
BD vs BVMT	< 0.001	0.666
BD vs NAB	0.007	0.594
BD vs JLO	< 0.001	0.900
VMI vs VP	0.065	0.401
VMI vs BVMT	< 0.001	0.755
VMI vs NAB	0.014	0.538
VMI vs JLO	< 0.001	0.778
VP vs BVMT	0.194	0.120
VP vs NAB	0.614	0.120
VP vs JLO	0.047	0.439
BVMT vs NAB	0.128	0.458
BVMT vs JLO	< 0.001	0.750
JLO vs NAB	0.002	0.641

*Note*. Standard Scores used here.

## Data Availability

The datasets used and/or analyzed during the current study are available from the corresponding author upon reasonable request.

## Abbreviations

BD: Block Design
BVMT: Brief Visuospatial Memory Test
DCCS: Dimensional Change Card Sort
Flanker: Flanker Inhibitory Control and Attention
FSIQ: Full Scale Intelligence Quotient
H3K4me3: histone H3 lysine 4 trimethylation
ICC: Intraclass Correlation Coefficient
IQ: Intelligence Quotient
JLO: Judgment of Line Orientation
KMT2D: lysine methyltransferase 2D
KS: Kabuki syndrome
KS1: Kabuki syndrome type 1
KS-CAB: Kabuki syndrome cognitive assessment battery
MDEM: Mendelian disorder of the epigenetic machinery
MIM: Mendelian Inheritance in Man
NIH: National Institutes of Health
NIHTB-CB: NIH Toolbox Cognitive Battery
NAB-SHL: Neuropsychological Assessment Battery Shape Learning
PSM: Picture Sequence Memory
VMI: Visual-Motor Integration
VP: Visual Perception
WAIS-IV: Wechsler Adult Intelligence Scale–Fourth Edition
WISC-IV: Wechsler Intelligence Scale for Children–Fourth Edition
